# Serum Cortisol and Clinical Response to a Single Session of Whole-Body Vibration in Healthy Adult Dogs

**DOI:** 10.3389/fvets.2021.563898

**Published:** 2021-02-01

**Authors:** Filipe C. I. Tannus, Sheila C. Rahal, Eunice Oba, Miriam Tsunemi, Bruna M. Silva, Karina C. Almeida, Ivan F. C. Santos

**Affiliations:** ^1^School of Veterinary Medicine and Animal Science, São Paulo State University (UNESP), Botucatu, Brazil; ^2^Department of Veterinary Surgery and Animal Reproduction, School of Veterinary Medicine and Animal Science, São Paulo State University (UNESP), Botucatu, Brazil; ^3^Department of Biostatistics, Bioscience Institute, São Paulo State University (UNESP), Botucatu, Brazil

**Keywords:** vibrating platform, hormone, stress, mechanic vibrations, systolic blood pressure

## Abstract

This study evaluated the serum cortisol response to a single session of whole-body vibration (WBV) in healthy adult dogs. Ten healthy adult medium dogs, females and males, aged between 24 and 48 months and with body weight between 10.1 and 17.9 kg were used. A single WBV session at a frequency of 30 Hz for 5 min (3.10 mm peak displacement, 11.16 m/s^2^ peak acceleration, and 0.29 m/s velocity), then 50 Hz for 5 min (3.98 mm peak displacement, 39.75 m/s^2^ peak acceleration, and 0.62 m/s velocity), and finishing with 30 Hz for 5 min (3.10 mm peak displacement, 11.16 m/s^2^ peak acceleration, and 0.29 m/s velocity) was performed. Serum cortisol, heart and respiratory rate, and systolic blood pressure were evaluated at different time points: 1 min before WBV (1PRE) and 1 min (1POST), 60 min (60POST), and 360 min (360POST) after the WBV session. An increase (*P* = 0.0417) of the serum cortisol values was observed between 1PRE and 1POST and a decrease (*P* = 0.0417) between 1POST and 60POST and between 60POST and 360POST. However, the values remained within the reference range. The heart and respiratory rate and the systolic blood pressure remained unchanged. Our findings suggest that a single bout of WBV (5 min of 30 and 50 Hz) using a vibrating platform that delivered a vortex wave circulation does not modify the serum cortisol levels and clinical parameters of healthy adult dogs.

## Introduction

Whole-body vibration (WBV) is a mechanical vibration stimulus that propagates throughout the body by using vibrating platforms (1, 2). Whole-body vibration has been used in human patients as an alternative modality of physical activity performed on vibrating platforms (1–5). The vibrating platform technology produces movements along the *x, y*, and *z* planes, and vortex wave circulation (3) and has been used to treat injuries and other debilitating conditions in human patients to strengthen and increase muscle mass and to improve cardiovascular functions (1, 5, 6).

The WBV effects are related to neuromuscular system responses (2) and that muscles are capable of detecting vibrations through mechanical stimuli transfer (7). Positive outcomes can likely be obtained by using WBV as a rehabilitation modality in animals. These positive outcomes, such as increased muscle mass, can improve the stability of joints affected by a degenerative disease (elbow and hip dysplasia) since these conditions can induce muscle atrophy (8).

Amplitude, frequency, WBV duration, the position of the animals on the vibrating platforms, the type of vibrating platforms, heterogeneity of patients, and the patient's age are some variables that influence the results (1, 2, 6). Thus, controlled studies must be carried out to obtain better scientific evidence and to establish effectiveness and safe protocols (1, 2). Few studies have evaluated the effect of WBV on dogs (8–11). A study showed that the renal resistivity index was not altered in healthy dogs subjected to a single WBV session (5 min with 30, 50, and 30 Hz) (9). Another study pointed out that the hematology, serum biochemistry, leg muscle thermography, and femoral resistive index of healthy adult Beagle dogs were not adversely affected by the WBV sessions performed for five consecutive days (8, 9). A report case showed the influence of WBV at the spontaneous opening of the cervix in a female dog with purulent metritis after a single session on the vibrating platform based on the same vibration protocol used in the literature (12). A study conducted by Nagai et al. (11) on healthy adult dogs has not identified alterations in the renal and common carotid resistive index after a single session of WBV (30 and 50 Hz for 15 min) using the vibrating platform which delivered a vortex wave circulation.

Hormonal responses after the WBV session have been evaluated in some studies with human patients, but their results are conflicting (2, 13, 14). Cortisol can stimulate bone reabsorption or inhibit bone formation (2), and it increases protein degradation and decreases protein synthesis in muscle cells, a fact that results in catabolism, especially in type II (15). Therefore, it is necessary to understand the importance of WBV to serum cortisol, mainly when it comes to skeletal mass maintenance (2), and the acute and chronic changes in cortisol blood concentration are often examined during exercise (15).

Since WBV can be used as a physical activity modality, it is important to evaluate serum cortisol by taking into account that cortisol can have negative effects on muscle mass (2). The present study aimed to evaluate the serum cortisol and clinical parameters' response to a single session of WBV in healthy adult dogs by using a vibrating platform that delivered a vortex wave circulation as mechanical vibration. The hypothesis was that a single session of WBV in healthy adult dogs (5 min with 30, 50, and 30 Hz) will decrease the serum cortisol and will not influence negatively the clinical parameters.

## Materials and Methods

This study was approved by the Ethics Committee for the Use of Animals of School of Veterinary Medicine and Animal Science, Botucatu, São Paulo State University (Unesp), no. 043/2016.

### Experimental Environment and Animal Selection

The study was conducted during the winter months—June and July, at GPS coordinates −22.8833 latitude and −48.4833 longitude. The experimental procedures were explained to the dogs' owners, and they signed a form allowing to use the animals in the study. All animals had a controlled diet (dry food) and used the same brand of food and were non-athlete dogs.

Fifteen healthy non-spayed and non-neutered dogs were recruited, and only 10 dogs (three females and seven males) were included in the study, aged 36 ± 8 months [mean ± standard deviation (SD)] and with the following morphometric measurements (mean ± SD): body weight (BW) of 14.3 ± 2.7 kg, body length (BL) of 41.4 ± 3.3 cm, and forelimb height (FH) of 34.4 ± 1.2 cm. The body proportion (BP) was smaller than 1 and indicated dogs with a long body or short limbs.

The inclusion criteria were the absence of disease or abnormalities in the physical examination, as well as normal complete blood count (CBC), serum biochemistry values, and urinalysis records. In addition, only female dogs at anestrus were selected for the study. The estrus cycle stage of the females was performed by vaginal cytology. The exclusion criteria were dogs that had not undergone any surgery or that were not treated with any medication in the 6 months prior to it and dogs that had not recorded any score lower than 4 or higher than 5 in the nine-point body condition scoring (16). Five dogs were excluded from the study since there were observed changes in the CBC and serum biochemistry values.

The BW was measured by using a pet digital scale 200 kg/100 g (70 × 50) (1) (FCIT, KCA). Fiberglass body tape was used to measure the FH and BL. BL was the distance from the cranial aspect of the shoulder joint to the caudal aspect of the sciatic tuberosity, and FH was the distance from the ground to the dorsal scapular rim. BP was calculated using the following formula: BP = FH/BL (FCIT, BMS). Dogs with BP below 1 were considered to have a long body or short limbs, and a BP above 1 would indicate a dog with a short body or long limbs (16).

### Whole-Body Vibration

The WBV session was performed at 07:00 a.m., after an 8-h overnight fasting. Dogs were subjected to 1-h acclimation before the WBV session on top of the vibrating platform (disconnected), and the room temperature was kept at 22°C and humidity was set between 35 and 40%. A maximum of three persons with minimum movement in the room was allowed, and noise was avoided. Previous training to use the vibrating platform was not necessary.

Dogs were positioned in a standing position (FCIT, BMS, KCA) in the center of the vibrating platform^1^ (92 cm length, 62 cm width, and 16 cm height) designed for dogs and cats, with protocol as previously described in the literature (8–11): single WBV session at a vibration frequency of 30 Hz for 5 min (3.10 mm peak displacement, 11.16 m/s^2^ peak acceleration, and 0.29 m/s velocity), then 50 Hz for 5 min (3.98 mm peak displacement, 39.75 m/s^2^ peak acceleration, and 0.62 m/s velocity), and finishing with 30 Hz for 5 min (3.10 mm peak displacement, 11.16 m/s^2^ peak acceleration, and 0.29 m/s velocity). According to the manufacturer, the vibration frequencies can be chosen between 05 and 100 Hz, and a vortex wave circulation was the mechanical vibration delivered by this platform. The frequency was checked by using a Digital Oscilloscope.^2^ A three-axis digital accelerometer sensor^3^ placed at the center of the vibrating platform was used for acquisition of the peak acceleration (*A*_peak_). Peak displacement (*D*_peak_) was calculated by using: *D*_peak_ = *A*_peak_/(2. *f* )^2^, *f* being the frequency (6) (KCA). The velocity (*V*) was determined by the following formula: *V* = *D*_peak_. π. *f* (π = 3.14) (6) (KCA).

Dogs remained standing at the top of the vibrating platform, and they were prevented to sit and walk by being physically restrained. The researcher's hand was positioned in the dog's ventral abdomen and removed after the dog stands up, and walking was avoided by means of a leash.

Clinical parameter evaluation and blood samples for serum cortisol evaluation were performed in the same room and condition where the WBV session was performed. Serum cortisol and clinical parameters were measured at 1 min before the WBV (1PRE) and at 1 min (1POST), 60 min (60POST), and 360 min (360POST) after the WBV session.

### Clinical Parameters

Clinical parameters included the respiratory rate (RR), heart rate (HR), and systolic blood pressure (SBP). All clinical parameters were measured (FCIT, BMS) with the dogs standing on the top of the vibrating platform in order to avoid stress and were performed by the same person. RR was measured by observation of costoabdominal respiration movements and HR by chest auscultation using a Classic III stethoscope.^4^ SBP was measured by using a Veterinary Doppler DVT 500^5^ with a Doppler transducer placed in the metacarpal artery.

### Serum Cortisol

Three milliliters of blood samples was obtained from the jugular vein for serum cortisol evaluation (BMS). Blood samples were collected with a 21-gauge needle on a 5-ml syringe with the dogs standing on the vibrating platform. Blood samples were placed immediately in a 5-ml plastic tube without coagulant and centrifuged after 30 min, 5 min at 4,000 U/min for 10 min.

Serum cortisol samples were stored at −80°C for 2 days and the values were assayed based on the radioimmunoassay methodology with commercial kits.^6^ The serum was incubated in tubes coated with a monoclonal antibody and added with ^125^I-bound tracers to cortisol. The liquid content in the tubes was aspirated and radioactivity binding was measured. The calibration curve was established, and unknown values were determined through standard curve interpolation. The curve provided the cortisol concentrations in the samples, which were measured at the same time with the calibrators. Results were calculated based on the semi-logarithmic curve (spline mode)—*B*/*T* (in percent) or *B*/*B*_0_ (in percent) was on the vertical axis and the cortisol concentrations of the calibrators were on the horizontal axis (in nanomoles per liter) (EO, FCIT). The reference range established by our laboratory for healthy dogs was 6.5–46.4 ng/ml.

### Animal Behavior Evaluation

A dog's behavior was evaluated before, during, and at 360 min after the WBV session by using the visualization method. Information regarding food and water intake, the presence of diarrhea or vomiting, and animal behavior 24 h after the WBV session were also evaluated. The evaluations were performed by the same person.

### Statistical Analysis

Statistical analyses were performed in software R version 3.4.4 (2018-03-15) (MT). The Kolmogorov–Smirnov test was employed to evaluate the normal distribution of variables (CBC, ALT, CR, CK, and serum cortisol). The Friedman test was utilized to compare the medians of the variables among time points. Differences were considered significant at *P* < 0.05.

## Results

### Animal Behavior Evaluation

The dogs did not require training standing on the vibrating platform, and they presented good acceptance of the WBV session at a frequency of 30 Hz, although they tried to sit when the frequency increased from 30 to 50 Hz. There were no changes in food and water intake and presence of diarrhea or vomiting 24 h after the WBV session.

### Clinical Parameters and Serum Cortisol

The RR, HR, and SBP values did not show significant differences between time points (Table 1). Serum cortisol values showed an increase (*P* = 0.0417) between 1PRE and 1POST and a decrease (*P* = 0.0417) between 1POST and 60POST and between 60POST and 360POST (Table 2).

**Table 1:** Values of respiratory rate (RR), heart rate (HR), and systolic blood pressure (SBP) obtained 1 min before (1PRE) and 1 min (1POST), 60 min (60POST), and 360 min (360POST) after the end of the whole-body vibration (WBV) session in 10 healthy adult dogs.

**Parameters**	**IPRE (median ± IQR)**	**1POST (median ± IQR)**	**60POST (median ± IQR)**	**360POST (median ± IQR)**	***P*-values**
RR (mpm)	35.0 ± 1.8	36.0 ± 2.0	30.0 ± 1.8	22.5 ± 2.5	0.981
HR (bpm)	70.0 ± 2.3	74.5 ± 4.5	64.5 ± 4.8	62.0 ± 2.0	0.110
SBP (mmHg)	125.0 ± 25.2	140.0 ± 22.5	110.0 ± 17.5	106.0 ± 10.0	0.176

RR normal range: 18.0–36.0 mpm; HR normal range: 60.0–160.0 bpm; SBP normal range: 85.0–133.0 mmHg.

*IQR, interquartile range*.

**Table 2:** Values of serum cortisol (in nanograms per milliliter) obtained 1 min before (1PRE) and 1 min (1POST), 60 min (60POST), and 360 min (360POST) after the end of the WBV session in 10 healthy adult dogs.

**Time points**				
**1PRE (median ± IQR)**	**1POST (median ± IQR)**	**60POST (median ± IQR)**	**360POST (median ± IQR)**	***P-*value**
20.0 ± 5.8D^a^	30.5 ± 10.9C^a^	16.5 ± 8.3B^a^	10.0 ± 6.2A^a^	0.0417

Reference values: 6.5–46.4 ng/ml.

a *Statistic variations: A < B < C > D (P <0.0417)*.

## Discussion

The present study evaluated whether a single session of WBV cannot influence the serum cortisol and clinical parameters in healthy adult dogs. The results have confirmed the hypothesis that a single bout of WBV does not interfere negatively in these parameters based on the adopted protocol, even though the serum cortisol values showed significant variations between the evaluated time points, but all these values remained within the reference ranges.

Morphometric data showed a body proportion below 1, considering all dogs used in the study were with similar body conformation (17) since different body conformations can modify the distribution of vibrations throughout the body (6).

The vibrating platform used in the present study was designed specifically for animals, which avoided changes that could influence the results. This vibrating platform produces a vortex wave circulation that induces a micro-impact in joints, differentiating from other platforms that generate movements along the *x* and *y* planes and results in asynchronous movements (6). The authors believe that this difference can influence the intensity of the distribution of mechanical vibrations throughout the body and improve the response of the neuromuscular and cardiorespiratory systems (1, 2, 6). The discussion over which type of vibrating platform is better is currently confusing (6).

The WBV protocol used in the present study was the same one applied to medium-sized dogs in previous studies (8–11). These studies reported a slight discomfort when the vibrating frequency increased from 30 to 50 Hz, similar to the current study (8–10). Paraesthesia in the hind limbs and discomfort in the hip region in untrained individuals exposed to acute WBV were observed in the literature (16).

The RR, HR, and SBP values did not show differences among the time points and showed the same trend of variations between time points as compared with the serum cortisol values. Acute elevation in cortisol levels has been considered as part of the process to remodel muscle tissues; however, chronic high cortisol levels have been associated with adverse effects (15). Both plasmatic adrenocorticotropic hormone (ACTH) and cortisol were shown to be indicators of the stressors, such as restraint or vibration, and the ACTH reached its levels before the cortisol (15, 19). In the present study, only the serum cortisol was evaluated due to the acquisitive lack of plasmatic ACTH during the study.

Elevated cortisol levels have been used as a physiological indicator of stress in dogs (18, 19); however, studies with human patients have associated acute bouts of resistance exercising with significant increases in the cortisol and adrenocorticotropic hormone levels (15, 19). A study conducted in healthy men did not show changes in the serum cortisol after 25 min of WBV (20). On the other hand, a decrease in cortisol in human patients has been related to low-intensity exercises and an increase with high-intensity exercises (21).

A circadian pattern could be associated with a decrease in serum cortisol values, but was excluded because of the standardized blood sampling protocol. A study performed in clinically healthy horses submitted to a single session of WBV (vibration frequency of 15–21 Hz, 10 min) identified a decrease in serum cortisol values 10 min after the WBV session (22). Another study evaluated the effect of vibrating frequencies of 5 and 50 Hz in sinusoidal WBV (2 and 4 h daily, for 5 days) on the cortisol levels of adult male guinea pigs (23) and identified decreased cortisol levels at a frequency of 50 Hz. In human patients, a decrease of the cortisol values was observed when exposed to an acute session of vertical sinusoidal WBV, 10 times for 60 s, with rest of 60 s between the vibration sets, and using a frequency of 26 Hz (peak displacement = 4 mm) (14), and after a single session of WBV with the same frequency and 26 Hz and peak displacement of 1 mm (24). This outcome was attributed to the possible inhibitory influence on the hypothalamic neurosecretory centers (14); WBV should not indicate catabolic activities in and/or negative effects on brain functions in older individuals. Regarding small animals, Blazizza et al. (25) used the same WBV protocol as in the present study to evaluate the variations of serum cortisol in healthy adult cats after being submitted to a single WBV. These authors did not observe significant differences in serum cortisol immediately after and at 4 h after the end of the WBV session, different from our study where a significant increase of the serum cortisol values was observed between 1 min before and 1 min after the WBV session, and a decrease after 60 and 360 min.

Creatine kinase could have been measured as a biomarker of muscle injury; however, a study conducted by Santos et al. (10) demonstrated that young healthy medium-sized dogs submitted to the same vibration protocol as used in the present study for five consecutive days did not show significant variations in the creatine kinase parameter.

The higher values of RR, HR, SBP, and serum cortisol observed prior to the study than those collected at 360POST were not imperative because all values were within the reference range before the WBV session. On the other hand, the WBV session was performed at the same time of the day, and the dogs were subjected to 1-h acclimation before the WBV session on top of the vibrating platform (disconnected). The room temperature and humidity were kept constant, and in all sessions, a number over three persons and noise were avoided.

The data in the present study suggest that a single bout of intermittent WBV exposure for 15 min was not a stressful stimulus for the neuroendocrine system of young dogs. The authors of the present study believe that the response of the clinical parameters due to a single session of WBV in healthy adult dogs was characterized by a pattern of hormonal secretion and can be typical of responses to acute mechanical vibrations. On the other hand, older individuals did not show significant differences regarding cortisol levels between immediately after and at 1 and 2 h after the single WBV session (30 Hz, 5 min) (13), which could be related to the lowest vibration frequency and exposure time on the vibrating platform.

Comparing the present study with studies in human subjects, it was possible to observe that numerous protocols have been used for different goals, and the comparison among studies in this research field is sometimes difficult because of the different protocols of WBV. Thus, further controlled studies should be performed to obtain better scientific evidence and to establish effective and safe protocols for WBV in dogs, which many consider as one of the limitations of the present study. Another limitation of this study was the use of a small sample of animals and no control group (dogs that stood on the vibrating platform turned off). The study was also conducted in young healthy dogs, so it is necessary to conduct studies that assess the consequences of WBV in older dogs with joint or muscular problems.

The clinical relevance observed in this study was associated with a possible decrease of the serum cortisol values after a single session of WBV in adult dogs by using a vibrating platform that produced a vortex wave circulation.

## Conclusions

Our findings suggest that a single bout of WBV (5 min of 30 and 50 Hz) by using a vibrating platform that delivered a vortex wave circulation does not modify the serum cortisol levels, respiratory rate, heart rate, and systolic blood pressure in healthy adult dogs.

## Data Availability Statement

The raw data supporting the conclusions of this article will be made available by the authors, without undue reservation.

## Ethics Statement

The animal study was reviewed and approved by Ethics Committee for the Use of Animals of School of Veterinary Medicine and Animal Science, Botucatu, São Paulo State University (Unesp). Written informed consent was obtained from the owners for the participation of their animals in this study.

## Author Contributions

IS, FT, and SR contributed to conception and design of the study. EO, KA, and BS performed Whole-body vibration session and measured clinical parameters, serum cortisol, and animal behavior. IS and FT wrote the original draft of this manuscript. All authors contributed to manuscript revision, read, and approved the submitted version.

## Conflict of Interest

The authors declare that the research was conducted in the absence of any commercial or financial relationships that could be construed as a potential conflict of interest.

## Footnotes

^1^TheraPlate Revolution®, TX, USA

^2^UNI-T UTD2102e®, USA

^3^LSM6DSM Accelerometer®, STMicroelectronics, São Paulo, Brazil

^4^3M™ Littmann® Stethoscopes, USA

^5^Martec Med®, Ribeirão Preto, São Paulo, Brazil

^6^Cortisol Ria kit®, Beckman Coulter, CA, USA
